# Embracing the Unprecedented Pace of Change: Artificial Intelligence's Impact on Dentistry and Beyond

**DOI:** 10.1055/s-0043-1770913

**Published:** 2023-07-20

**Authors:** Hakan Turkkahraman

**Affiliations:** 1 Department of Orthodontics and Oral Facial Genetics, School of Dentistry, Indiana University, Indianapolis, Indiana, United States

## Introduction

Human beings have long exhibited a natural fear of the unknown. We tend to initially ignore novel changes, only to gradually adapt to them as they become familiar. However, the recent emergence of generative artificial intelligence (AI) applications has propelled us into an era of technological advancement comparable to the invention of electricity. The remarkable speed of these developments has caught many off guard, surpassing our expectations. It is not the change itself that instills fear but rather the breathtaking pace at which it unfolds. The challenge lies in ensuring that regulations keep pace with technological advancements to guide the responsible use of AI. In this guest editorial, we explore the transformative potential of AI in dentistry and emphasize the need to embrace change while maintaining ethical frameworks.

## The Dawn of an Artificial Intelligence Revolution

AI has already revolutionized various aspects of our lives, from how we live and consume media to how we drive vehicles. Now, it stands poised to redefine every facet of our existence. As health care practitioners, we find ourselves on the precipice of significant transformations in the way we diagnose and treat patients. Educators, too must adapt their teaching methods to accommodate the changing landscape, as AI has already reshaped how students learn. For researchers, AI offers new avenues for conducting studies and generating insights. The inevitability of change urges us to reflect on whether we are prepared to abandon old habits and embrace the potential of AI in dentistry and health care as a whole.

## A Paradigm Shift in Diagnosis and Treatment

AI's impact on dentistry is profound, particularly in the realm of diagnosis and treatment. The ability of AI algorithms to analyze vast amounts of patient data, such as medical records, radiographs, and clinical images, holds tremendous promise. AI-powered image recognition systems can now detect and classify dental pathologies with unprecedented accuracy. 1 2 3 4 By augmenting the diagnostic process, AI empowers dentists to make informed decisions and devise personalized treatment plans, ultimately enhancing patient outcomes. 5 6 7 8 9 However, as we embark on this transformative journey, we must address concerns surrounding patient privacy, data security, and the responsible use of AI-generated insights.

## Empowering Education and Research

The integration of AI in dentistry also extends to education and research. Educational institutions must adapt their curricula to equip future dental professionals with the necessary skills to leverage AI technologies effectively. AI-based platforms and virtual simulations offer immersive learning experiences and facilitate knowledge acquisition. 10 11 12 Furthermore, researchers can harness AI's capabilities to analyze vast datasets, uncover patterns, and accelerate the pace of discovery. However, as we leverage AI's potential, we must ensure that ethical considerations and human oversight remain integral to the research process. Transparency, accountability, and avoiding biases in algorithm design are vital aspects that warrant attention.

## Regulatory Challenges and Ethical Imperatives

The remarkable speed of AI's advancement has outpaced the development of regulatory frameworks. It is imperative that we address this imbalance to prevent potential misuse and protect patient welfare. Striking the right balance between innovation and responsible deployment of AI requires the formulation of clear guidelines and robust ethical standards. Data protection, privacy, and informed consent are paramount considerations. Transparent and explainable AI algorithms should be developed to facilitate trust and ensure accountability. Collaboration between regulatory bodies, health care professionals, and technology experts is vital in navigating the complex landscape of AI in dentistry.

## Embracing Change for Optimal Patient Care

As dentists, researchers, educators, and health care professionals, we must proactively embrace change rather than resist it. AI presents a unique opportunity to enhance patient care, improve treatment outcomes, and streamline dental practice. By embracing AI's potential and actively contributing to the development of ethical frameworks, we can harness its transformative power for the benefit of both practitioners and patients. As we navigate this uncharted territory, we should strive to strike a balance between AI-driven innovation and maintaining the fundamental principles of human expertise, empathy, and patient-centered care.

## Conclusion

The unparalleled pace of change brought about by AI requires us to adapt swiftly to ensure its responsible integration into dentistry and health care. Embracing AI's potential in diagnosis, treatment planning, education, and research will revolutionize dental practice. However, it is crucial to address regulatory challenges, develop ethical frameworks, and safeguard patient privacy and welfare. The European Journal of Dentistry encourages ongoing research, dialogue, and collaboration to shape the future of AI in dentistry, ultimately fostering innovation while upholding the highest ethical standards.

## References

[1] Mertens S Krois J Cantu A G Arsiwala L T Schwendicke F Artificial intelligence for caries detection: randomized trial J Dent 2021 115 103849 34656656 10.1016/j.jdent.2021.103849

[2] Zhou X Yu G Yin Q Liu Y Zhang Z Sun J Context aware convolutional neural network for children caries diagnosis on dental panoramic radiographs Comput Math Methods Med 2022 2022 6.029245E6 10.1155/2022/6029245 PMC9519291 36188109

[3] Schwendicke F Cejudo Grano de Oro J Garcia Cantu A Meyer-Lueckel H Chaurasia A Krois J Artificial intelligence for caries detection: value of data and information J Dent Res 2022 101 11 1350 1356 35996332 10.1177/00220345221113756 PMC9516598

[4] Lee K S Kwak H J Oh J M Automated detection of TMJ osteoarthritis based on artificial intelligence J Dent Res 2020 99 12 1363 1367 32609562 10.1177/0022034520936950

[5] Mason T Kelly K M Eckert G Dean J A Dundar M M Turkkahraman H A machine learning model for orthodontic extraction/non-extraction decision in a racially and ethnically diverse patient population Int Orthod 2023 21 03 100759 37196482 10.1016/j.ortho.2023.100759

[6] Lee H Ahmad S Frazier M Dundar M M Turkkahraman H A novel machine learning model for class III surgery decisionJ Orofac Orthop2022 10.1007/s00056-022-00421-7 PMC11186927 36018345

[7] Leavitt L Volovic J Steinhauer L Can we predict orthodontic extraction patterns by using machine learning?Orthod Craniofac Res2023 10.1111/ocr.12641 36843547

[8] Mureșanu S Almășan O Hedeșiu M Dioșan L Dinu C Jacobs R Artificial intelligence models for clinical usage in dentistry with a focus on dentomaxillofacial CBCT: a systematic review Oral Radiol 2023 39 01 18 40 36269515 10.1007/s11282-022-00660-9

[9] Wood T Anigbo J O Eckert G Stewart K T Dundar M M Turkkahraman H Prediction of the post-pubertal mandibular length and y axis of growth by using various machine learning techniques: a retrospective longitudinal study Diagnostics (Basel) 2023 13 09 1553 37174945 10.3390/diagnostics13091553 PMC10178146

[10] Mahrous A Botsko D L Elgreatly A Tsujimoto A Qian F Schneider G B The use of artificial intelligence and game-based learning in removable partial denture design: a comparative studyJ Dent Educ2023 10.1002/jdd.13225 37186466

[11] Islam N M Laughter L Sadid-Zadeh R Adopting artificial intelligence in dental education: a model for academic leadership and innovation J Dent Educ 2022 86 11 1545 1551 35781809 10.1002/jdd.13010

[12] Imran E Adanir N Khurshid Z Significance of haptic and virtual reality simulation (VRS) in the dental education: a review of literature Appl Sci (Basel) 2021 11 21 10196

